# Intrinsically Fluorescent Oligomeric Cytotoxic Conjugates Toxic for FGFR1-Overproducing Cancers

**DOI:** 10.1021/acs.biomac.1c01280

**Published:** 2021-12-02

**Authors:** Natalia Porębska, Agata Knapik, Marta Poźniak, Mateusz Adam Krzyścik, Małgorzata Zakrzewska, Jacek Otlewski, Łukasz Opaliński

**Affiliations:** Faculty of Biotechnology, Department of Protein Engineering, University of Wroclaw, Joliot-Curie 14a, Wroclaw 50-383, Poland

## Abstract

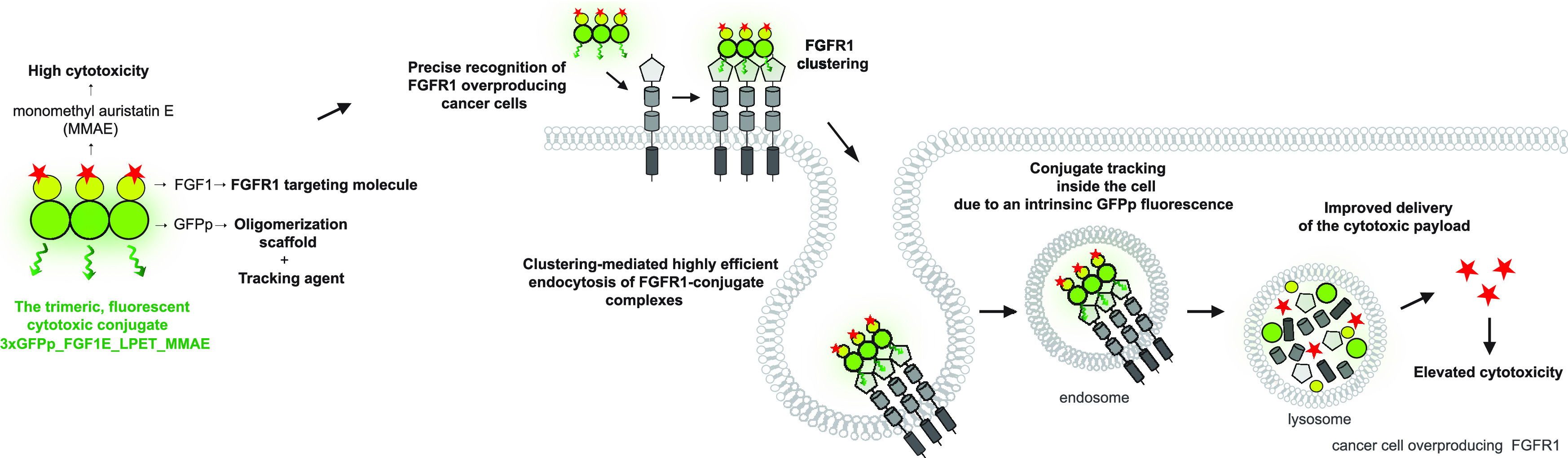

Fibroblast growth factor receptor 1 (FGFR1) is an integral membrane protein that transmits prolife signals through the plasma membrane. Overexpression of FGFR1 has been reported in various tumor types, and therefore, this receptor constitutes an attractive molecular target for selective anticancer therapies. Here, we present a novel system for generation of intrinsically fluorescent, self-assembling, oligomeric cytotoxic conjugates with high affinity and efficient internalization targeting FGFR1. In our approach, we employed FGF1 as an FGFR1 recognizing molecule and genetically fused it to green fluorescent protein polygons (GFPp), a fluorescent oligomerization scaffold, resulting in a set of GFPp_FGF1 oligomers with largely improved receptor binding. To validate the applicability of using GFPp_FGF1 oligomers as cancer probes and drug carriers in targeted therapy of cancers with aberrant FGFR1, we selected a trimeric variant from generated GFPp_FGF1 oligomers and further engineered it by introducing FGF1-stabilizing mutations and by incorporating the cytotoxic drug monomethyl auristatin E (MMAE) in a site-specific manner. The resulting intrinsically fluorescent, trimeric cytotoxic conjugate 3xGFPp_FGF1E_LPET_MMAE exhibits nanomolar affinity for the receptor and very high stability. Notably, the intrinsic fluorescence of 3xGFPp_FGF1E_LPET_MMAE allows for tracking the cellular transport of the conjugate, demonstrating that 3xGFPp_FGF1E_LPET_MMAE is efficiently and selectively internalized into cells expressing FGFR1. Importantly, we show that 3xGFPp_FGF1E_LPET_MMAE displays very high cytotoxicity against a panel of different cancer cells overproducing FGFR1 while remaining neutral toward cells devoid of FGFR1 expression. Our data implicate that the engineered fluorescent conjugates can be used for imaging and targeted therapy of FGFR1-overproducing cancers.

## 1 Introduction

Cancer is one of the leading causes of mortality worldwide.^1^ Conventional chemotherapy is currently the most commonly used cancer treatment approach, but unfortunately, due to nonspecific drug targeting and high toxicity, it also affects normal cells and generates numerous side effects.^1−3^ One of the most promising strategies in cancer treatment is targeted therapy with cytotoxic conjugates.^4^ The major advantage of this targeted approach is selective and precise delivery of the cytotoxic drug into tumors while avoiding normal cells and minimizing side effects of the therapy.^1,3−5^ This approach relies on the presence of specific macromolecules on the tumor surface, which are not produced at all or are present at very low levels on normal cells.^6^ Engineered targeting molecules, such as monoclonal antibodies or modified ligands, recognize cancer-specific macromolecules and utilize receptor-mediated endocytosis to deliver a cytotoxic payload into cancer cells, leading to their death. Among the many different types of cancer biomarkers, membrane receptors like growth factor receptors are predominant.^7,8^

Fibroblast growth factor receptor 1 (FGFR1) is a receptor tyrosine kinase (RTK) that, together with the extracellular fibroblast growth factors (FGFs), is involved in transmission of signals across the plasma membrane.^9−11^ FGFR1-dependent signaling regulates various biological processes like cell migration, proliferation, and apoptosis.^12^ Aberrant activity of this receptor causes many developmental disorders and is detected in numerous cancers.^13−16^ Overexpression of FGFR1 has been observed in various tumor types, like lung, breast, ovarian, prostate, head, and neck cancers.^17−20^ FGFR1 exposes a large region to the extracellular space, providing potential binding sites for targeting molecules.^20−22^ FGFR1 is very efficiently internalized by several endocytic pathways, and thus, its endocytosis can be hijacked for rapid intracellular drug delivery.^20^ Importantly, complexes of ligands/targeting molecules with FGFR1 avoid the unfavorable recycling pathway and are predominantly sorted to lysosomes for degradation and cytotoxic drug release.^20^ All these features make FGFR1 an attractive molecular target for selective anticancer therapies. To date, a few cytotoxic conjugates with antibody fragments or natural ligands as targeting molecules have been developed for the selective treatment of FGFR1-overproducing tumors.^23−28^ However, novel FGFR1-targeting molecules are still urgently needed to improve the efficiency and selectivity of drug delivery and to enable simultaneous visualization of the conjugates during their action.

We have recently shown that the high affinity of targeting molecules promotes their cellular uptake by FGFR1-dependent endocytosis.^29^ Furthermore, we have demonstrated that FGFR1 endocytosis is controlled by the spatial distribution of the receptor in the plasma membrane.^30−32^ The oligomeric ligand-induced FGFR1 clustering on the cell surface enhances the efficiency and simultaneously alters the mechanism of receptor endocytosis.^29^ Based on these findings, we have developed a novel system for generation of self-assembling, oligomeric drug carriers targeting FGFR1, which combine high affinity for FGFR1 and receptor clustering activity, ensuring precise recognition of the receptor on the cancer cell surface and highly efficient and selective drug delivery into the cancer cell interior. Additionally, we have equipped our oligomeric drug carriers with a stable intrinsic fluorescence for their monitoring. We demonstrate the applicability of our oligomeric drug carriers for efficient and selective deterioration of FGFR1-overproducing cancer cells by constructing a highly potent trimeric cytotoxic conjugate fused with monomethyl auristatin E.

## 2 Materials and Methods

### 2.1 Antibodies and Reagents

The primary antibodies directed against FGFR1 (#9740), phospho-FGFR (p-FGFR, #3476), ERK1/2 (#9102), and phospho-ERK1/2 (p-ERK1/2, #9101) were from Cell Signaling (Danvers, MA, USA). The antitubulin primary antibody (#T6557) was from Sigma-Aldrich (St. Louis, MO, USA). The anti-FGF1 primary antibody (#sc-55520) was from Santa Cruz Biotechnology (Dallas, TX, USA). HRP-conjugated secondary antibodies were obtained from Jackson Immuno-Research Laboratories (Cambridge, UK).

Reagents used for the solid-phase peptide synthesis were as follows: amino Fmoc-Gly-OH, Fmoc-l-Cys(StBu)–OH, Fmoc-O2Oc-O2Oc–OH; COMU (1-[1-(cyano-2-ethoxy-2-oxoethylideneaminooxy)-dimethylamino-morpholino]uroniumhexafluorophosphate), EDT (ethane-1,2-dithiol), piperidine, TIS (triisopropylsilane), DIPEA (*N*,*N*-diisopropylethylamine), DMF (*N*,*N*-dimethylformamide), DCM (dichloromethane), and TFA (trifluoroacetic acid) were purchased from Iris Biotech GmbH (Marktredwitz, Germany). HPLC-pure acetonitrile and Et_2_O (diethyl ether) were from Avantor (Gliwice, Poland). TentaGel S RAM resin (particle size, 90 μm; loading, 0.21 mmol/g) was from Rapp Polymere GmbH (Tübingen, Germany). The cytotoxic agents, MMAE (monomethyl auristatin E) and MC-vc-PAB–MMAE, were from MedChemExpress (Monmouth Junction, NJ, USA). A Synergi 4 μm Fusion-RP 80 Å 250 × 10 mm^2^ LC column was from Phenomenex, Inc.

### 2.2 Cells

Mouse embryo fibroblast cells (NIH3T3) were obtained from American Type Culture Collection (ATCC, Manassas, VA, USA). NIH3T3 were cultured in Dulbecco’s modified Eagle’s medium (DMEM) (Thermo Fisher Scientific, Waltham, MA, USA) supplemented with 10% fetal bovine serum (Thermo Fisher Scientific, Waltham, MA, USA) and antibiotics (100 U/mL penicillin and 100 μg/mL streptomycin). The human osteosarcoma cell line (U2OS) was purchased from American Type Culture Collection (ATCC, Manassas, VA, USA), and U2OS cells stably expressing FGFR1 (U2OS-R1) were obtained by transfection of U2OS cells with expression plasmids encoding FGFR1.^23^ U2OS cells were cultivated in DMEM (Biowest, Nuaille, France) supplemented with 10% fetal bovine serum (Thermo Fisher Scientific, Waltham, MA, USA) and antibiotics (100 U/mL penicillin and 100 μg/mL streptomycin). For U2OSR1 cells, growth media were additionally supplemented with geneticin (0.5 mg/mL) (Thermo Fisher Scientific, Waltham, MA, USA). The human lung cancer cell line NCI-H520, the breast cancer cell line NCI-H1581, and the osteosarcoma cell line G-292 were from ATCC (Manassas, VA, USA). HCC-15 cells (human squamous cell lung carcinoma) were obtained from the Leibniz Institute DSMZ, German Collection of Microorganisms and Cell Cultures. The NCI-H520 cell line was cultivated in an RPMI 1640 medium (ATCC) supplemented with 10% fetal bovine serum and antibiotics (100 U/mL penicillin and 100 μg/mL streptomycin). NCI-H1581 and HCC-15 cell lines were cultured in an RPMI 1640 medium (Biowest) supplemented with 10% fetal bovine serum (Thermo Fisher Scientific, Waltham, MA, USA) and antibiotics (100 U/mL penicillin and 100 μg/mL streptomycin). The G-292 cell line was cultured in DMEM (Biowest, Nuaille, France) supplemented with 10% fetal bovine serum (Thermo Fisher Scientific, Waltham, MA, USA) and antibiotics (100 U/mL penicillin and 100 μg/mL streptomycin). All cell lines were cultured in a 5% CO_2_ atmosphere at 37 °C and were seeded onto tissue culture plates one day prior to the start of the experiments.

### 2.3 Recombinant Proteins

The plasmid pET28a_HisTag-GFPpoly_protG was a kind gift from the Jung lab, Department of Chemistry, National University in Daejeon, South Korea.^33^ To obtain genetic constructs for expression of GFPp_FGF1, the protG sequence was exchanged for an FGF1 sequence using the restriction free cloning technique. The GFPp_FGF1 oligomers were expressed in an *Escherichia coli* (*E. coli*) BL21(DE3)-RIL strain (Agilent Technologies, Santa Clara, CA, USA). Cells were grown at 37 °C until OD_600_ = 0.8. Protein expression was induced by addition of 0.5 mM IPTG followed by incubation of cells at 16 °C for 16 h. GFPp_FGF1 oligomers were purified by affinity chromatography using a HiTrap heparin column (GE Healthcare, Piscataway, NJ, USA). Various oligomeric forms (from monomers to tetramers) were isolated via elution from the column with a NaCl gradient (in 25 mM HEPES, pH 7.6) in a range from 0.2 to 2 M using an NGC chromatography system (Bio-Rad, Hercules, CA, USA). The purity and the identity of the obtained oligomers were confirmed by SDS-PAGE, Western blotting, and native PAGE.

A genetic construct designed for production of 3xGFPp_FGF1E_LPETGG was prepared to enable site-specific conjugation of the cytotoxic drug to GFPp_FGF1E via sortase A-mediated ligation.^34−36^ To obtain this construct, we used a mutant variant of FGF1, FGF1E, with three mutations stabilizing the protein structure (Q40P, S47I, and H93G) and three cysteines exchanged to serine residues (C16S, C83S, and C117S).^25^ The construct with an introduced C-terminal LPETGG sequence, GFPp_FGF1E_LPETGG, was prepared via gene synthesis. The protein was expressed in a bacterial system and purified with heparin affinity chromatography as described above.

An evolved sortase A (eSortA) pentamutant with improved kinetics and activity was produced in an *E. coli* strain as described earlier.^37,38^

The wild-type FGF1 and the extracellular region of FGFR1 fused to the Fc fragment of human IgG1 were produced as described previously.^39,40^

### 2.4 Synthesis of GGGG-PEG4-vcMMAE

As a first step, the H_2_N-GGGG-PEG4-C-CONH_2_ peptide was synthesized by solid-phase peptide synthesis (SPPS) in the Fmoc strategy. The peptide was hydrolyzed from the resin with a mixture of TFA/EDT/TIS/H_2_O (vol %, 95:2:2:1), triply precipitated in cold Et_2_O, purified by reverse-phase high-performance liquid chromatography (RP-HPLC), and lyophilized. H_2_N-GGGG-PEG4-C-CONH_2_ (38 mg, 57 μmol) and maleimide-vcMMAE (MC-vc-PAB–MMAE, 25 mg, 19 μmol, 0.3 equiv) were then dissolved in 1000 μL of DMAc followed by the addition of DIPEA (30 μL, 171 μmol, 3 equiv). The reaction was conducted at 30 °C for 12 h. The solvent was then removed under vacuum, and the GGGG-PEG4-vcMMAE was purified by RP-HPLC and lyophilized. The identity of the product was confirmed by MALDI-MS.

### 2.5 Native Page

Separation of proteins under nondenaturing conditions was performed using native PAGE.^41^ Proteins (5 μg) were separated on 10% native gels using Tris-glycine running buffer (25 mM Tris, 192 mM glycine, pH 8.3). Native gels were run on ice, at 100 V, and after separation, gels were imaged under UV light or stained with CBB.

### 2.6 FGFR1 Activation

To analyze the impact of oligomeric variants of GFPp_FGF1 on the activation of FGFR1 and initiation of receptor-downstream signaling cascades, serum-starved NIH3T3 cells (12-well plates, 100,000 cells/well) were treated with increasing concentrations of the wild-type FGF1 or GFPp_FGF1 oligomers (0.1, 0.5, 1, and 2 ng/mL) in the presence of heparin (10 U/mL) for 15 min at 37 °C (the concentrations of all GFPp_FGF1 oligomers were normalized to the molar concentration of FGF1 WT). Cells were lysed in Laemmli buffer and subjected to SDS-PAGE and Western blotting. The experiment was performed analogously for 3xGFPp_FGF1E_LPETGG and 3xGFPp_FGF1E_LPET_MMAE with a protein concentration of 2 ng/mL.

To study the kinetics of FGFR1 activation, serum-starved NIH3T3 cells (12-well plates, 100,000 cells/well) were incubated with GFPp_FGF1 oligomers (20 ng/mL) in the presence of heparin for 6 h. At distinct time points (15 min, 30 min, and 1, 2, 4, and 6 h), cells were lysed in Laemmli buffer and analyzed by SDS-PAGE and Western blotting.

### 2.7 BLI Measurements

Binding analysis of oligomeric variants of GFPp_FGF1 to FGFR1ecd-Fc was performed using biolayer interferometry (BLI) with ForteBio Octet K2 (Pall ForteBio, San Jose, CA, USA). FGFR1-Fc (10 μg/mL) was immobilized on Protein A sensors, and association and dissociation phases were monitored at various concentrations of GFPp_FGF1 oligomers (75, 150, 300, and 600 nM) in PBS buffer. A reference sensor without FGFR1ecd-Fc was used as a control. Kinetic parameters of the interaction were determined based on a global 2:1 “heterogeneous ligand” fitting with ForteBio Data Analysis 11.0 software (Pall ForteBio, San Jose, CA, USA). The experiment was performed analogously for 3xGFPp_FGF1E_LPETGG and 3xGFPp_FGF1E_LPET_MMAE.

### 2.8 Conjugation of the Trimeric GFPp_FGF1E_LPETGG with MMAE via Sortase A-Mediated Ligation

The protocol for the conjugation of GFPp_FGF1E_LPETGG with MMAE via sortase A-mediated ligation was done according to protocols established previously by us, with optimization regarding the concentration of sortase A (0.1–0.5 μM) and peptide-MMAE (10–200 μM).^37^ The purified engineered trimeric 3xGFPp_FGF1E_LPETGG protein containing a C-terminal LPETGG sequence was transferred to the sortase A reaction buffer (25 mM HEPES at pH 7.6, 154 mM NaCl, 5 mM CaCl_2_, and 2 mM TCEP) using HiTrap desalting columns (Thermo Fisher Scientific, Waltham, MA, USA). The final concentration of the protein used in the conjugation reaction was 400 μg/mL. The GGGG-PEG_4_-vcMMAE peptide was added to the protein solution to a final concentration of 100 μM. Then, sortase A was added to a final concentration of 0.1 μM, and the mixture was incubated for 12 h at 15 °C. After incubation, the protein was purified by affinity chromatography using a HiTrap heparin column (GE Healthcare, Piscataway, NJ, USA). The resin was washed with washing buffer containing 25 mM HEPES at pH 8.0, 0.2 M NaCl, 1 mM EDTA, and 1 mM DTT to remove unconjugated MMAE, and then, 3xGFPp_FGF1E_LPET_MMAE was eluted with elution buffer containing 25 mM HEPES at pH 8.0, 2 M NaCl, 1 mM EDTA, and 1 mM DTT. The efficiency of the conjugation was confirmed by SDS-PAGE. The identity of the conjugate was confirmed by MALDI-MS.

### 2.9 Analysis of Protein Stability

To analyze the stability of 3xGFPp_FGF1E_LPETGG and 3xGFPp_FGF1E_LPET_MMAE, the protein and the conjugate (20 μg) were incubated in 10-fold diluted human serum (Sigma-Aldrich, St. Louis, MO, USA) in the presence of 10 U/mL heparin at 37 °C for 96 h. At distinct time points (0, 24, 48, 72, and 96 h), samples were taken, and proteins were analyzed with native PAGE, SDS-PAGE, and Western blotting.

In addition, the stability of the protein and the conjugate was analyzed by measuring their biological activity after incubation in human serum. NIH3T3 cells were cultured on the 12-well plates (100,000 cells/well) in a serum-free medium for 24 h. Next, proteins (taken at time points 0, 24, 48, 72, and 96 h of incubation in human serum) were added to the media to a final concentration of 100 ng/mL in the presence of heparin (10 U/mL) and incubated with cells for 15 min at 37 °C. Cells were lysed in Laemmli buffer, and activation of cell signaling cascades was analyzed by SDS-PAGE and Western blotting.

The stability of the protein and the conjugate was also analyzed by measuring their fluorescence. The concentration of proteins was 1 μM; proteins were incubated up to 96 h in 10-fold diluted human serum. Fluorescence spectra were acquired using an FP-8500 spectrofluorometer (Jasco, Japan) with excitation at 488 nm and emission in the 500–650 nm range.

### 2.10 Fluorescence Microscopy

For the analysis of FGFR1 dependence of 3xGFPp_FGF1E_LPETGG and 3xGFPp_FGF1E_LPET_MMAE cellular uptake, early endosomes in U2OS-R1 cells were stained with Rab5a-RFP (CellLight Early Endosomes-RFP, Thermo Fisher Scientific, Waltham, MA, USA), according to the manufacturer’s protocol. Then, U2OS-R1 cells were seeded on the plate with an equal number of nonstained U2OS cells and left to attach overnight. Cells were preincubated with recombinant proteins (5 μg/mL) in a serum-free medium supplemented with 10 U/mL heparin for 30 min on ice, and next, cells were transferred to 37 °C and incubated for 45 min. After that time, the internalization was stopped by cooling down of cells on ice. Cells were subsequently washed with PBS, nuclei were stained with a NucBlue Live dye (Thermo Fisher Scientific, Waltham, MA, USA), and cells were fixed in 4% paraformaldehyde solution.

To analyze the internalization kinetics of 3xGFPp_FGF1E_LPETGG and 3xGFPp_FGF1E_LPET_MMAE into cells expressing FGFR1, early endosomes in U2OS-R1 cells were labeled with Rab5a-RFP (CellLight Early Endosomes-RFP). Cells were preincubated with proteins (5 μg/mL) in the presence of heparin for 30 min on ice. Then, cells were transferred to 37 °C, and incubation was continued for 5, 15, or 45 min. After incubation, the internalization was stopped by cooling down of cells on ice. Next, cells were washed with PBS, nuclei were labeled with a NucBlue Live dye, and cells were fixed. Wide-field fluorescence microscopy was carried out using a Zeiss Axio Observer Z1 fluorescence microscope (Zeiss, Oberkochen, Germany). Images were taken using an LD-Plan-Neofluar 40×/0.6 Korr M27 objective and an Axiocam 503 camera. The fluorescence of proteins was visualized with a 450/490 nm bandpass excitation filter and a 500/550 nm bandpass emission filter. The CellLight Early Endosomes-RFP signal was visualized with a 540/552 nm bandpass excitation filter and a 575/640 nm bandpass emission filter. The NucBlue Live signal was visualized with a 335/383 nm bandpass excitation filter and a 420/470 nm emission filter. Images were processed with Zeiss ZEN 2.3 software (Zeiss, Oberkochen, Germany) and Adobe Photoshop (Adobe, San Jose, CA, USA).

### 2.11 Flow Cytometry Analysis

U2OS and U2OS-R1 (100,000 cells/well) cells were seeded onto 12-well plates in a full medium and left to attach overnight. Then, the medium was removed, and cells were washed with PBS buffer and starved with a serum-free medium for 4 h. Next, plates were cooled on ice, and 3xGFPp_FGF1E_LPETGG and 3xGFPp_FGF1E_LPET_MMAE (500 ng/mL) were added to the cells in a serum-free medium supplemented with 1% BSA and heparin (10 U/mL). After 30 min of incubation on ice, cells were transferred to 37 °C for 30 min to allow for internalization. Then, the medium was removed, and cells were washed with cold PBS (three times, 2 min). Next, cells were washed with a serum-free medium supplemented with 0.2% BSA, pH = 3.5 (three times, 5 min), and then with PBS buffer (three times, 1 min) and detached by 10 mM EDTA in PBS, pH 8.0. Cells were subsequently harvested by centrifugation, resuspended in PBS with 1% BSA, and analyzed by flow cytometry using a NovoCyte 2060R flow cytometer and NovoExpress software (ACEA Biosciences, San Diego, CA, USA).

### 2.12 FGFR1 Degradation

To analyze FGFR1 degradation kinetics, serum-starved U2OS-R1 cells (12-well plates, 100,000 cells/well) were treated with cycloheximide (10 μg/mL), FGF1 WT, 3xGFPp_FGF1E_LPETGG, and 3xGFPp_FGF1E_LPET_MMAE (20 ng/mL) in the presence of heparin (10 U/mL) for 12 h at 37 °C (the concentrations of recombinant proteins were normalized to the molar concentration of FGF1 WT). At distinct time points (5 min and 1, 3, 6, and 12 h), cells were lysed in Laemmli buffer and analyzed by SDS-PAGE and Western blotting.

### 2.13 Cytotoxicity Assay

The cytotoxicity of the 3xGFPp_FGF1E_LPET_MMAE was tested on the FGFR1-negative cell line (HCC15) and FGFR1-positive cell lines (NCI-H520, NCI-H1581, and G-292). Cells in the appropriate full medium were plated at 5000 cells per well in 96-well plates and incubated overnight at 37 °C in the presence of 5% CO_2_. Cells were treated with increasing concentrations (from 0.01 to 100 nM) of 3xGFPp_FGF1E_LPETGG (negative control) and 3xGFPp_FGF1E_LPET_MMAE or the free drug (positive control) in the presence of heparin (10 U/mL) for 96 h at 37 °C. Next, cell viability was measured using a PrestoBlue cell viability reagent (Thermo Fisher Scientific, Waltham, MA, USA), according to the manufacturer’s protocol. Fluorescence emission at 590 nm (excitation at 560 nm), reflecting the viability of the cells, was measured using an Infinite M1000 PRO plate reader (Tecan, Männedorf, Switzerland). Statistical analyses were performed for three independent experiments using *t*-tests. EC_50_ values were calculated based on the Hill equation using Origin 7 software (Northampton, MA).

### 2.14 Mass Spectrometry

The molecular mass of the protein and the conjugate was determined by matrix-assisted laser desorption/ionization–time-of-flight–mass spectrometry (MALDI-TOF-MS, AB 4800+, Applied Biosystems, Waltham, MA) using sinapinic acid as the matrix.

## 3 Results

### 3.1 Oligomerization of FGF1 with GFPpolygons

**Figure 1:**
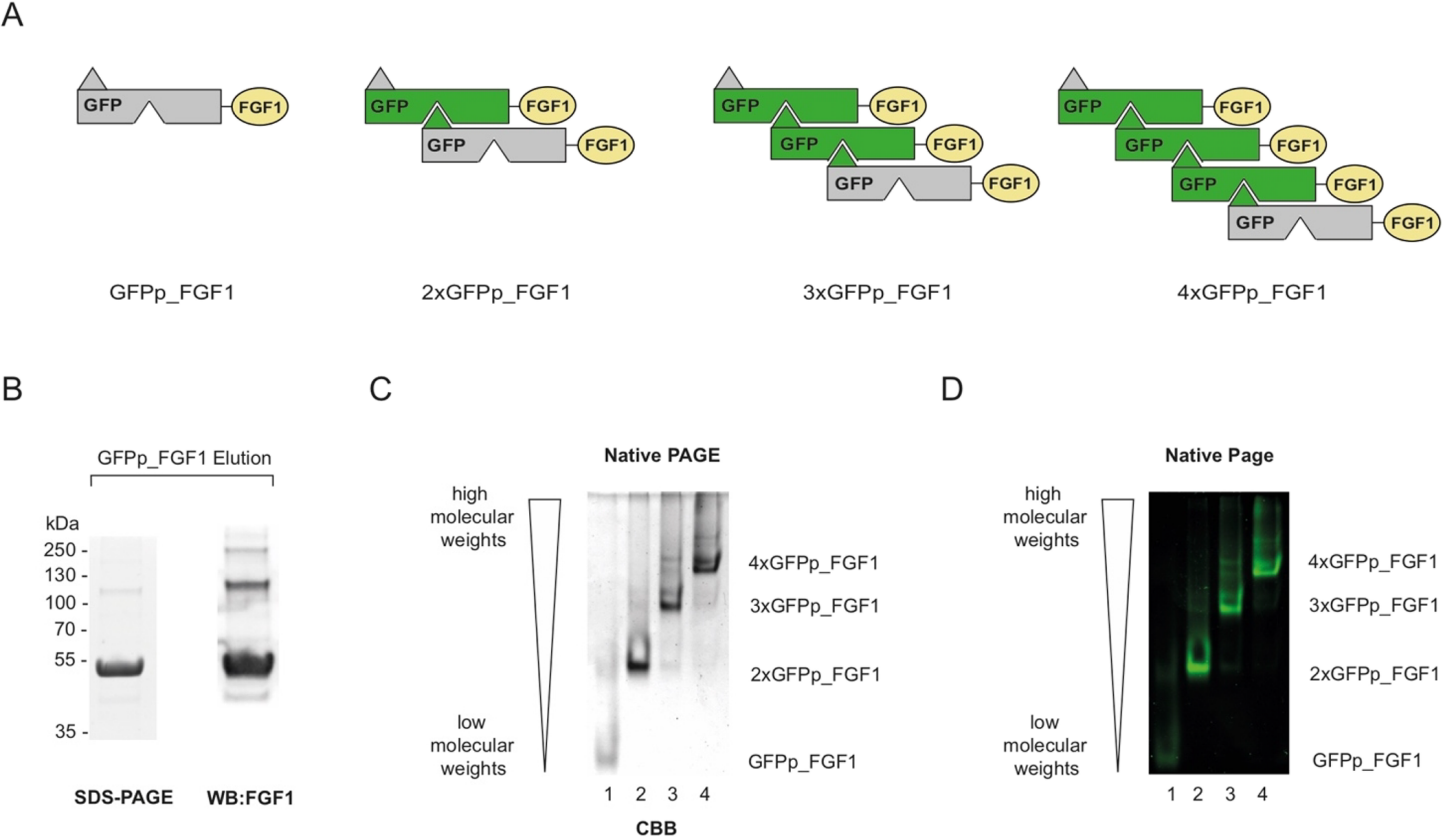
Preparation of GFPp_FGF1 oligomers. (A) Schematic representation of engineered oligomeric GFPp_FGF1 ligands. Nonfluorogenic monomeric GFPpolygons are labeled in gray, FGF1 is labeled in yellow, and fluorescent GFPpolygon-based FGF1 oligomers are marked in green. (B) The mixture of GFPp_FGF1 oligomers was purified by affinity chromatography and analyzed using SDS-PAGE and Western blotting with anti-FGF1 antibodies. (C) Various oligomeric forms were isolated via elution from the heparin Sepharose column with a NaCl gradient. The oligomeric state and the purity of the obtained GFPp_FGF1 fractions were confirmed by native PAGE (CBB staining). (D) Fluorescence properties of purified GFPp_FGF1 oligomers were assessed with UV light imaging of native PAGE gels; CBB, Coomasie Brilliant Blue.

We sought to construct a platform for the efficient generation of self-assembling, high-affinity, efficiently internalizing, oligomeric FGFR1-targeting molecules that could serve as drug delivery agents for the precise treatment of FGFR1-dependent cancers. Furthermore, we intended to develop a strategy in which molecules targeting FGFR1 would simultaneously exhibit an intrinsic fluorescence, enabling visualization of their trafficking in cells, organs, or even in the whole body. Therefore, we employed FGF1 as a high-affinity ligand of FGFR1 and genetically fused it to green fluorescent protein polygons (GFPp) for controlled oligomerization and fluorescence visualization. GFPp is a modified GFP variant in which one of the β-sheets has been transferred to another region of the protein. This prevents intramolecular folding of the GFP and formation of a fluorogenic β-barrel but provides intermolecular GFPp interactions that form fluorogenic variants with different oligomeric states (Figure 1A).^33^ By fusing FGF1 with GFPp, self-assembling GFPp_FGF1 variants of different oligomeric states can be obtained (Figure 1A). Importantly, in this approach, only oligomers of GFPp_FGF1 display intrinsic fluorescence, allowing for visualization of GFPp_FGF1 (Figure 1A).

GFPp_FGF1 was successfully expressed in a bacterial protein expression system, and the resulting mixture of different oligomeric variants of GFPp_FGF1 was purified by affinity chromatography. The purity and the identity of proteins were confirmed by SDS-PAGE and Western blotting using anti-FGF1 antibodies (Figure 1B). We observed the assembly of stable GFPp_FGF1 oligomers that were partially resistant to denaturing conditions (Figure 1B).

We developed a protocol for separation of various GFPp_FGF1 oligomeric forms from each other by using a heparin Sepharose column and elution with a NaCl gradient. Using this approach, we were able to obtain a highly pure GFPp_FGF1 monomer, dimer, trimer, and tetramer (Figure 1C). As expected, only GFPp_FGF1 oligomers displayed intrinsic fluorescence (Figure 1D).

### 3.2 GFPp_FGF1 Oligomers with Improved Binding to FGFR1

**Figure 2:**
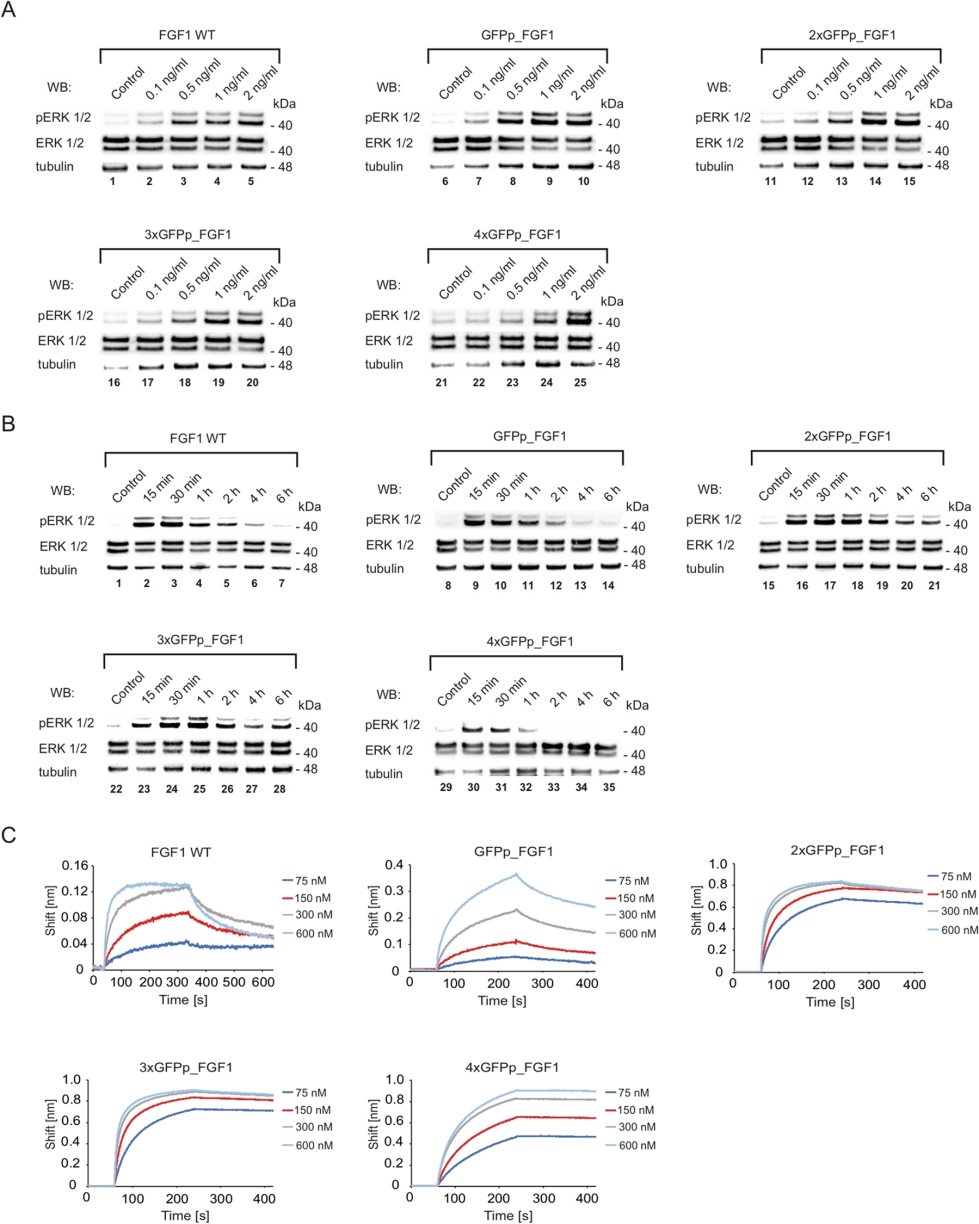
Impact of engineered GFPp_FGF1 oligomers on FGFR1 binding and activation. (A) Serum-starved NIH3T3 cells were treated with increasing concentrations of the wild-type FGF1 or GFPp_FGF1 oligomers. Cells were lysed, and activation of FGFR1 and receptor-downstream signaling were assessed with Western blotting. The level of tubulin served as a loading control. (B) To determine the kinetics of FGFR1 signaling upon cell stimulation with GFPp_FGF1, serum-starved NIH3T3 cells were stimulated with proteins for up to 6 h. At distinct time points of incubation, cells were lysed and analyzed by Western blotting. (C) Kinetics of the interaction of GFPp_FGF1 oligomers with FGFR1 was analyzed using biolayer interferometry (BLI). The extracellular region of FGFR1 (FGFR1ecd-Fc) was immobilized on Protein A sensors, and then, the receptor was incubated with distinct GFPp_FGF1 oligomers. The association and dissociation profiles were measured.

To investigate whether FGF1 within GFPp_FGF1 retained the ability to bind and activate FGFR1 and to analyze the impact of GFPp-mediated FGF1 oligomerization on FGFR1 activation and initiation of receptor-dependent signaling pathways, serum-starved NIH3T3 cells were treated with increasing concentrations of the wild-type FGF1 or GFPp_FGF1 oligomers. Cells were lysed and analyzed by Western blotting. As shown in Figure 2A, all obtained GFPp_FGF1 oligomers efficiently induced phosphorylation of ERK1/2 kinases in a concentration-dependent manner and to a similar extent to the wild-type FGF1.

In the next step, we analyzed the kinetics of FGFR1 signaling upon stimulation of cells with oligomeric proteins. At distinct time points of incubation with GFPp_FGF1 oligomers, cells were lysed and analyzed by Western blotting. As shown in Figure 2B, the dimeric 2xGFPp_FGF1 (lanes 15–21) and the trimeric 3xGFPp_FGF1 (lanes 22–28) displayed prolonged activation of the receptor in comparison to the wild-type FGF1. In contrast, the tetrameric 4xGFPp_FGF1 variant showed shorter activation of FGFR1 (lanes 29–35). The kinetics of the interaction of GFPp_FGF1 oligomers with FGFR1 was analyzed using biolayer interferometry (BLI). To this end, the extracellular region of FGFR1 (FGFR1ecd-Fc) was immobilized on Protein A sensors, and then, the receptor was incubated with distinct GFPp_FGF1 oligomers or the wild-type FGF1 as a control.

We observed that all recombinant proteins directly interacted with the receptor, as expected (Figure 2C). In addition, all GFPp_FGF1 oligomers showed largely improved binding to FGFR1 as compared to the monomeric wild-type FGF1 or the monomeric GFPp_FGF1 (Figure 2C and Table 1). Kinetic parameters revealed reduced dissociation rates of GFPp_FGF1 oligomers for FGFR1ecd-Fc (*k*_off_), indicating that oligomeric proteins formed a more stable complex with the receptor than the monomeric wild-type FGF1 (Table 1).

**Table 1:** Kinetic Parameters of the Interaction between GFPp_FGF1 Oligomers and FGFR1^a^

FGFR1ecd-Fc	*K*_D1_ [M]	*K*_D2_ [M]	*K*_on1_ [M^–1^ s^–1^]	*K*_on2_ [M^–1^ s^–1^]	*K*_Off1_ [s^–1^]	*K*_Off2_ [s^–1^]
FGF1 WT	3 × 10^–8^	4.8 × 10^–8^	6.73 × 10^4^	6.07 × 10^5^	2 × 10^–3^	2.91 × 10^–2^
GFPp_FGF1	1.91 × 10^–8^	6.21 × 10^–8^	1.73 × 10^4^	2.34 × 10^5^	3.31 × 10^–4^	1.45 × 10^–2^
2xGFPp_FGF1	2.34 × 10^–9^	1 × 10^–12^	5.03 × 10^5^	4.67 × 10^4^	1.18 × 10^–3^	1 × 10^–7^
3xGFPp_FGF1	1 × 10^–12^	1.48 × 10^–8^	5.32 × 10^5^	4.12 × 10^4^	1 × 10^–7^	6.1 × 10^–4^
4xGFPp_FGF1	1 × 10^–9^	1.4 × 10^–10^	1.9 × 10^4^	1.53 × 10^5^	2.14 × 10^–5^	2.14 × 10^–5^

a Measurements were conducted using a biolayer interferometry (BLI) technique. Parameters of the interaction were determined by global fitting with the 2:1 “heterogeneous ligand” with ForteBio Data Analysis 11.0 software.

All these data demonstrate that oligomeric GFPp_FGF1 variants efficiently bind and activate FGFR1. GFPp-mediated FGF1 oligomerization significantly improves FGFR1 binding and affects the kinetics of FGFR1 signaling. The discrepancies in the duration of signal propagation between distinct GFPp_FGF1 oligomers may arise from their differential architecture and affinity for the receptor. This generates diversity in the spatial organization of FGFR1, possibly affecting receptor kinase activity, endocytosis, and feedback regulatory pathways.

### 3.3 Engineering of the Trimeric Cytotoxic Conjugate Targeting FGFR1

**Figure 3:**
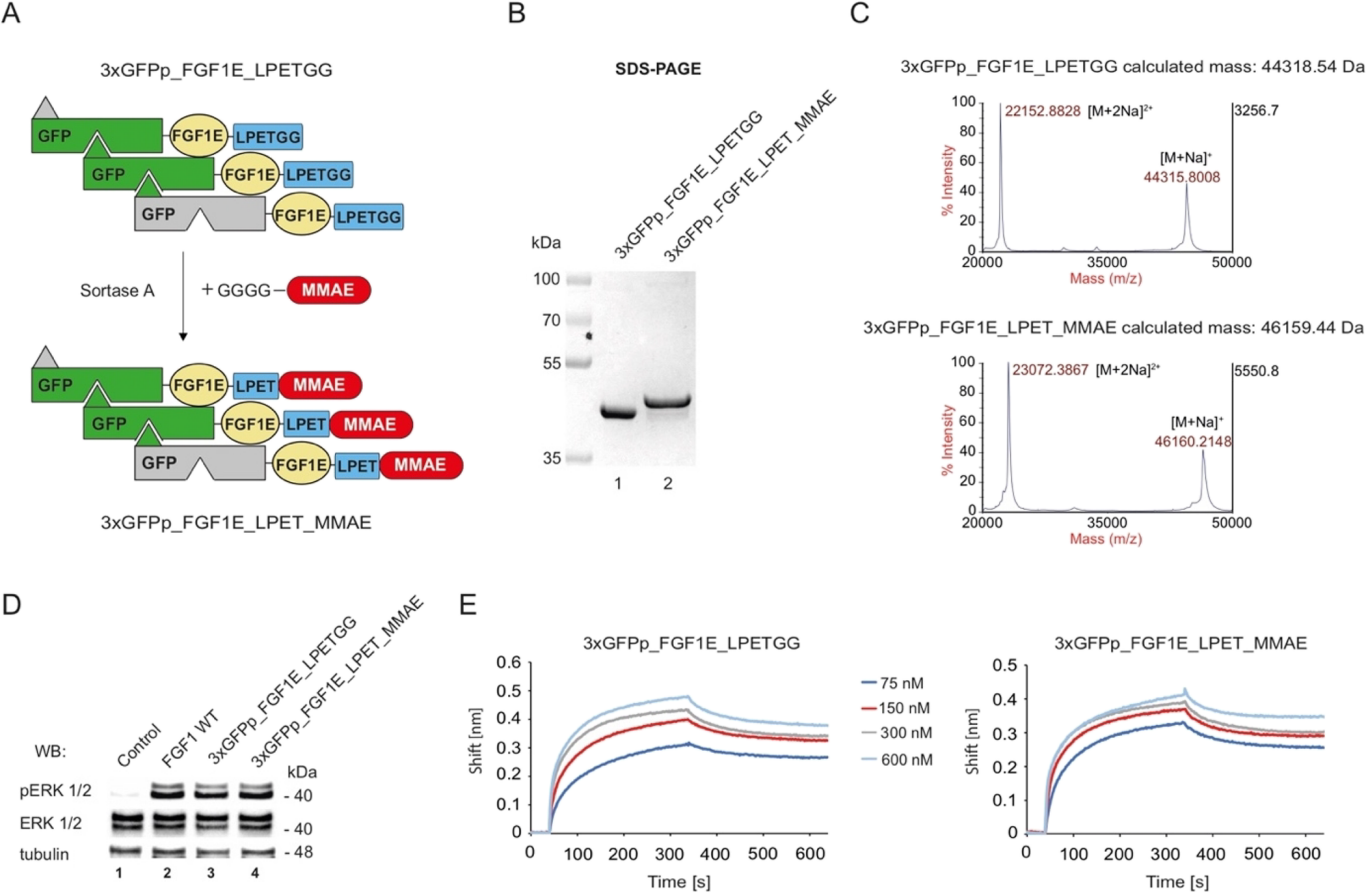
Engineering of the fluorescent trimeric cytotoxic conjugate targeting FGFR1. (A) The C-terminal LPETGG sequence was incorporated into the trimeric GFPp_FGF1E via gene synthesis, yielding 3xGFPp_FGF1E_LPETGG. Sortase A recognizes the LPETGG sequence within 3xGFPp_FGF1E_LPETGG and mediates ligation of the tetraglycine peptide-linked MMAE to 3xGFPp_FGF1E_LPETGG, resulting in 3xGFPp_FGF1E_LPET_MMAE. (B) The efficiency of the conjugation and purity of the obtained 3xGFPp_FGF1E_LPET_MMAE were confirmed by SDS-PAGE. (C) The site-specific attachment of MMAE to 3xGFPp_FGF1E_LPETGG was confirmed by MALDI-MS. The impurities that appear in MALDI-MS (about 30,000 Da) are either the result of a minor protein fragmentation during ionization or trace impurities/degradation products not visible in SDS-PAGE and UV spectra but detectable in the high-sensitivity MS approach. (D) Assessment of the biological activity of recombinant proteins. Serum-starved NIH3T3 cells were incubated with FGF1 WT (positive control) or with 3xGFPp_FGF1E_LPETGG and 3xGFPp_FGF1E_LPET_MMAE. Cells were lysed, and activation of FGFR1 was assessed with Western blotting. The level of tubulin served as a loading control. (E) Binding profiles of 3xGFPp_FGF1E_LPETGG and 3xGFPp_FGF1E_LPET_MMAE to FGFR1 were measured using BLI. The extracellular region of FGFR1 (FGFR1ecd-Fc) was immobilized on Protein A sensors and incubated with proteins/conjugates. Association and dissociation profiles were measured.

Based on FGFR1 binding characteristics, we selected from GFPp_FGF1 oligomers the trimeric variant to engineer an intrinsically fluorescent oligomeric cytotoxic conjugate targeting cancer cells overproducing FGFR1. To improve the stability of the cytotoxic conjugate, we decided to use the mutant variant of FGF1, FGF1E with three substitutions that stabilize the protein structure (Q40P, S47I, and H93G) and three cysteines exchanged to serines (C16S, C83S, and C117S) (resulting in *T*_den_ = 47 °C, about 7 °C higher than that of the wild-type protein), instead of the wild-type FGF1.^25^ To enable a site-specific conjugation of the cytotoxic drug (a potent tubulin-destabilizing agent, monomethyl auristatin E (MMAE), successfully used in conjugates approved for cancer treatment) to the trimeric GFPp_FGF1E, we incorporated a C-terminal LPETGG sequence to the protein, resulting in 3xGFPp_FGF1E_LPETGG (Figure 3A).^37,42,43^ Sortase A recognizes the LPETGG sequence within 3xGFPp_FGF1E_LPETGG and mediates site-specific ligation of the MMAE- linked tetraglycine peptide to 3xGFPp_FGF1E_LPETGG, resulting in the trimeric fluorogenic cytotoxic conjugate 3xGFPp_FGF1E_LPET_MMAE (Figure 3A).^34,35,37,44^ 3xGFPp_FGF1E_LPETGG was produced in a bacterial system, purified to homogeneity (Figure 3B, lane 1), and subsequently efficiently conjugated to MMAE via sortase A-mediated ligation, as evidenced by an alteration in migration on SDS-PAGE (Figure 3B, lane 2). Additionally, the site-specific attachment of MMAE to 3xGFPp_FGF1E_LPETGG was confirmed by MALDI-MS (Figure 3C).

To verify if changes introduced to the trimeric GFPp_FGF1 did not affect the proteins’ ability to interact with FGFR1, we analyzed the impact of 3xGFPp_FGF1E_LPETGG and the cytotoxic conjugate 3xGFPp_FGF1E_LPET_MMAE on FGFR1 activation and initiation of receptor-dependent signaling pathways. As shown in Figure 3D, both proteins tested efficiently induced phosphorylation of ERK1/2.

Next, we analyzed whether attachment of the cytotoxic drug affected the recognition of FGFR1 by FGF1 within the conjugate. Kinetic parameters revealed that the conjugate retained increased affinity for FGFR1, as did the nonconjugated trimeric 3xGFPp_FGF1E_LPETGG (Figure 3E) (Table 2).

**Table 2:** Kinetic Parameters of Interactions of the Trimeric Protein and Its Conjugate with FGFR1^a^

FGFR1-Fc	*K*_D1_ [M]	*K*_D2_ [M]	*K*_on1_ [M^–1^ s^–1^]	*K*_on2_ [M^–1^ s^–1^]	*K*_Off1_ [s^–1^]	*K*_Off2_ [s^–1^]
3xGFPp_FGF1E_LPETGG	1 × 10^–12^	5.97 × 10^–9^	2.58 × 10^4^	4.27 × 10^5^	1 × 10^–7^	2.55 × 10^–3^
3xGFPp_FGF1E_LPET_MMAE	1 × 10^–12^	2.91 × 10^–9^	2.56 × 10^4^	6.7 × 10^5^	1 × 10^–7^	2.04 × 10^–3^

a Measurements were conducted using a biolayer interferometry (BLI) technique. Parameters of the interaction were determined by global fitting with the 2:1 “heterogeneous ligand” with ForteBio Data Analysis 11.0 software.

All these data demonstrate the successful development of the trimeric, intrinsically fluorescent cytotoxic conjugate 3xGFPp_FGF1E_LPET_MMAE with high affinity for the cognate receptor.

### 3.4 High Stability of the Trimeric Cytotoxic Conjugate

**Figure 4:**
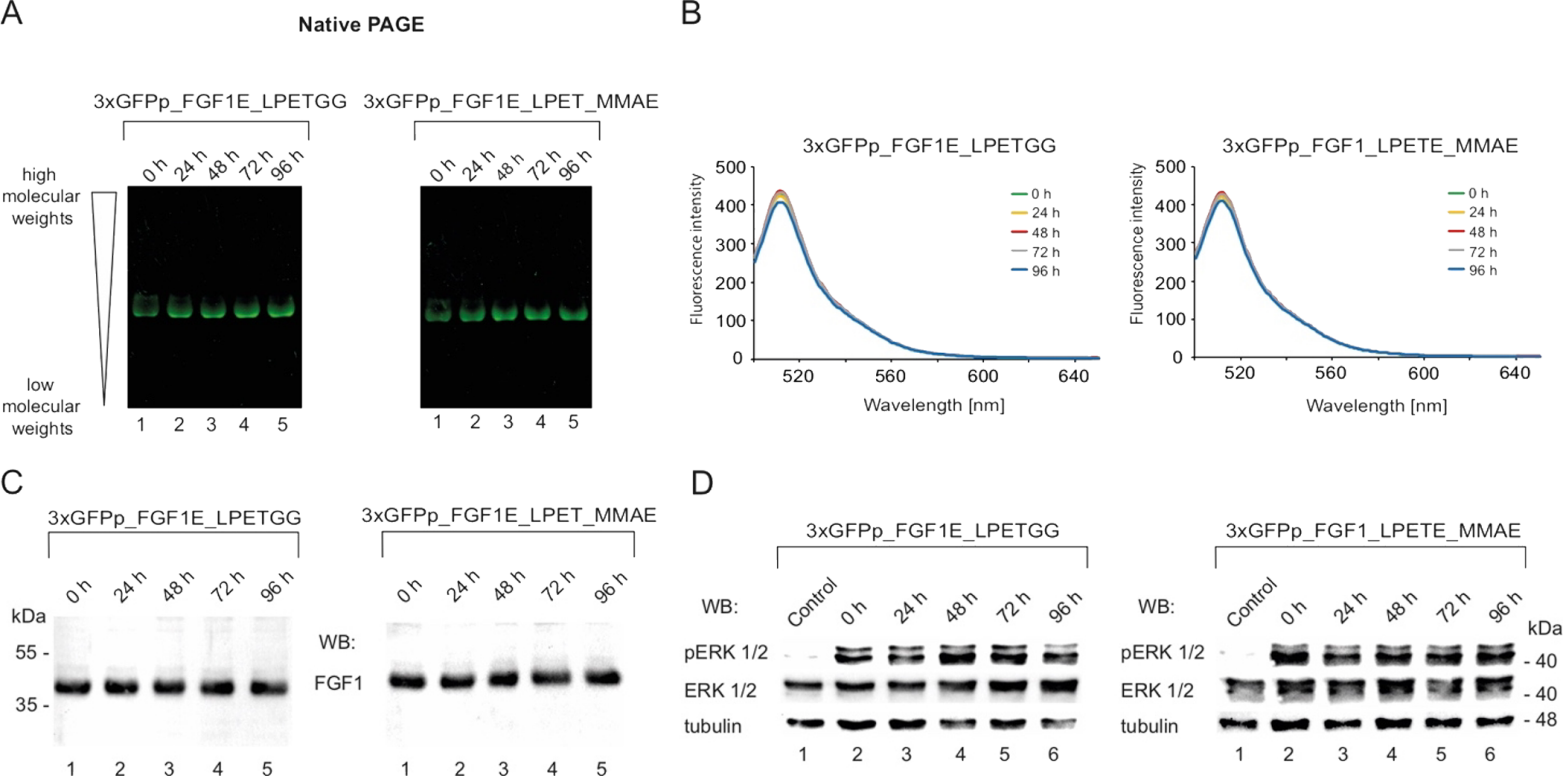
Stability analysis of the 3xGFPp_FGF1E_LPETGG and 3xGFPp_FGF1E_LPET_MMAE. (A) 3xGFPp_FGF1E_LPETGG and 3xGFPp_FGF1E_LPET_MMAE were incubated in human serum in the presence of heparin at 37 °C for 96 h. At distinct time points (0, 24, 48, 72, and 96 h), samples were taken, and the oligomeric state of proteins was analyzed using native PAGE UV light imaging. (B) The stability of the GFPp oligomerization scaffold within the trimeric protein and the conjugate was determined by monitoring GFP fluorescence at distinct time points of incubation in human serum at 37 °C. Fluorescence spectra were acquired using a FP-8500 spectrofluorometer (Jasco, Japan) with excitation at 488 nm and emission in the 500–650 nm range. (C) The stability of FGF1E in 3xGFPp_FGF1E_LPETGG and 3xGFPp_FGF1E_LPET_MMAE was determined with Western blotting using antibodies recognizing FGF1. (D) Evaluation of the biological activity of 3xGFPp_FGF1E_LPETGG and its cytotoxic conjugate. Samples were incubated with human serum at 37 °C for 96 h. At distinct time points of incubation (0, 24, 48, 72, and 96 h), proteins were added to serum-starved NIH3T3 cells. Cells were lysed, and activation of FGFR1 and receptor-downstream signaling were assessed with Western blotting. The level of tubulin served as a loading control.

In the next step, we analyzed the stability of the trimeric 3xGFPp_FGF1E_LPETGG and 3xGFPp_FGF1E_LPET_MMAE in human serum. Proteins were incubated in serum in the presence of heparin at 37 °C for 96 h. At distinct time points (0, 24, 48, 72, and 96 h), samples were taken, and the oligomeric state of the proteins was analyzed using native PAGE. As shown in Figure 4A, the proteins were very stable as their oligomeric state was virtually unchanged even after 96 h of incubation at 37 °C. We also made use of the natural fluorescence of GFPp molecules and determined the stability of the GFPp oligomerization scaffold within the trimeric protein and the conjugate by monitoring GFP fluorescence. As shown in Figure 4B, the fluorescence spectra of the trimeric protein and its conjugate did not change even after 96 h of incubation of 3xGFPp_FGF1E_LPETGG and 3xGFPp_FGF1E_LPET_MMAE in human serum.

The stability of FGF1E in the trimer and in the trimeric conjugate was determined with Western blotting using antibodies that recognize FGF1. We observed that the level of FGF1 in the oligomeric proteins was unaltered even after long-term incubation at 37 °C (Figure 4C).

To evaluate the biological activity of FGF1E within the trimeric variant and its conjugate upon prolonged incubation in human serum, induction of FGFR1-dependent signaling pathways by the trimeric protein and the conjugate was monitored using NIH3T3 cells. As shown in Figure 4D, both 3xGFPp_FGF1E_LPETGG and 3xGFPp_FGF1E_LPET_MMAE effectively induced phosphorylation of ERK1/2 kinases, even after 96 h of incubation.

All these data indicate that the drug vehicle 3xGFPp_FGF1E_LPETGG and the resulting cytotoxic conjugate 3xGFPp_FGF1E_LPET_MMAE are very stable and retain full FGFR1 binding capacity and biological activity even after long-term incubation in human serum.

### 3.5 Efficient Internalization of the Trimeric Conjugate into Cells Expressing FGFR1

The effectiveness of the anticancer therapy with cytotoxic conjugates largely relies on the selective delivery of toxic drugs into cancer cells. We have recently shown that FGFR1 clustering either with engineered multivalent antibodies or oligomeric ligands strongly enhances the efficiency and alters the mechanism of receptor endocytosis.^24,30,31^ We have also demonstrated that high affinity of FGFR1-specific antibodies promotes their uptake via receptor-mediated endocytosis.^24,29,31^ These results implied that the oligomeric cytotoxic conjugate 3xGFPp_FGF1E_LPET_MMAE, due to its very high affinity for the receptor and FGFR1 cross-linking potential, could serve as a highly efficient drug carrier for the treatment of FGFR1-overproducing cancers. Importantly, the intrinsic fluorescence of the 3xGFPp_FGF1E_LPET_MMAE should allow for precise monitoring of the conjugate trafficking. Thus, we analyzed the efficiency and FGFR1 dependence of internalization of the trimeric carrier protein 3xGFPp_FGF1E_LPETGG and its conjugate 3xGFPp_FGF1E_LPET_MMAE.

**Figure 5:**
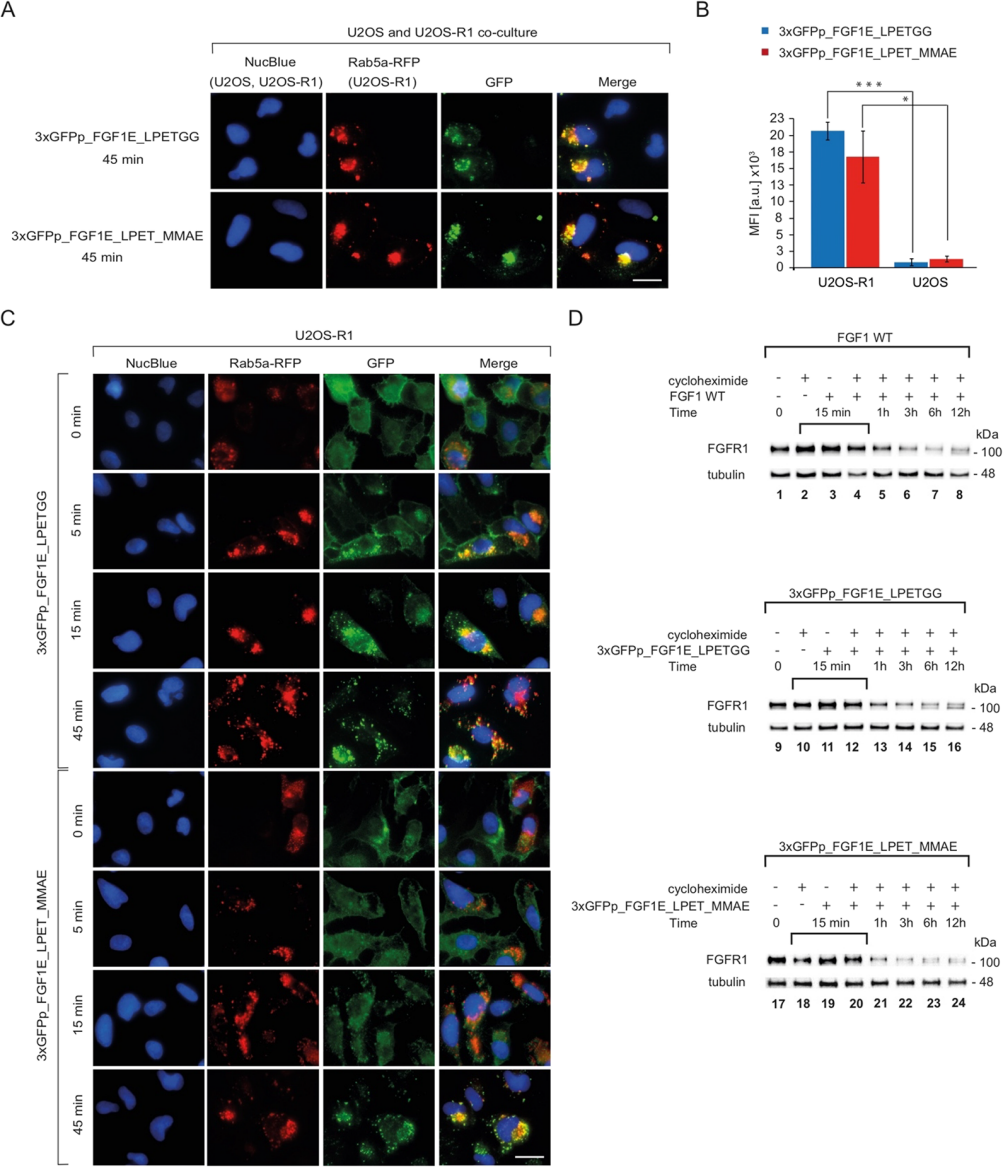
Internalization of fluorescent 3xGFPp_FGF1E_LPETGG and its conjugate into cells expressing FGFR1. (A) Equal numbers of U2OS-R1 cells prestained with Rab5a-RFP and nonstained U2OS cells were cocultured and preincubated at 4 °C with fluorescent proteins and transferred to 37 °C for 45 min. Nuclei were labeled with NucBlue Live, and cells were analyzed by confocal microscopy. The scale bar represents 50 μm. (B) The quantitative analysis of the cellular uptake of 3xGFPp_FGF1E_LPETGG and 3xGFPp_FGF1E_LPET_MMAE was performed using flow cytometry. Serum-starved cells were incubated with proteins on ice for 30 min. Then, cells were transferred to 37 °C for 30 min and subsequently analyzed by flow cytometry. The data shown are mean fluorescence intensities (MFI) from three independent experiments ± SD. Statistical significance: *p < 0.05, ^**^p < 0.01, and ^***^p < 0.001. (C) Kinetics of the internalization of 3xGFPp_FGF1E_LPETGG and 3xGFPp_FGF1E_LPET_MMAE into U2OS-R1 cells. Serum-starved U2OS-R1 cells prestained with Rab5a-RFP (red) were incubated on cold with fluorescent proteins (green) for 30 min and then transferred to 37 °C. At various time points, cells were fixed and analyzed with fluorescence microscopy. The scale bars correspond to 50 μm. (D) Kinetics of FGFR1 degradation upon stimulation with 3xGFPp_FGF1E_LPETGG and 3xGFPp_FGF1E_LPET_MMAE. Serum-starved U2OS-R1 cells were treated with cycloheximide to inhibit synthesis of new FGFR1 molecules and incubated with proteins for various time points. Cells were lysed, and the level of FGFR1 was determined with Western blotting. The level of tubulin served as a loading control.

To analyze whether internalization of the 3xGFPp_FGF1E_LPETGG and 3xGFPp_FGF1E_LPET_MMAE proteins occurs selectively via FGFR1-mediated endocytosis, we employed two model cell lines, U2OS cells lacking a detectable level of FGFR1 and U2OS cells stably transfected with FGFR1 (U2OS-R1). Early endosomes in U2OS-R1 cells were prestained with Rab5a-RFP. Then, U2OS-R1 cells were cocultured with nonstained U2OS cells, treated with 3xGFPp_FGF1E_LPETGG and 3xGFPp_FGF1E_LPET_MMAE, and analyzed with fluorescence microscopy. As shown in Figure 5A, colocalization of GFP and Rab5a-RFP signals was detected in U2OS-R1 cells for both 3xGFPp_FGF1E_LPETGG and 3xGFPp_FGF1E_LPET_MMAE, indicating efficient FGFR1-mediated endocytosis. Importantly, the fluorescence of 3xGFPp_FGF1E_LPETGG and 3xGFPp_FGF1E_LPET_MMAE was not observed in control U2OS cells (Figure 5A).

Additionally, we performed quantitative analysis of the cellular uptake of 3xGFPp_FGF1E_LPETGG and 3xGFPp_FGF1E_LPET_MMAE using flow cytometry. As shown in Figure 5B, 3xGFPp_FGF1E_LPETGG and 3xGFPp_FGF1E_LPET_MMAE were more than 10-fold more efficiently internalized into FGFR1-positive U2OS-R1 cells, as compared to the control U2OS cells.

Next, we analyzed the kinetics of internalization of 3xGFPp_FGF1E_LPETGG and 3xGFPp_FGF1E_LPET_MMAE into U2OS-R1 cells using fluorescence microscopy. At time point zero, 3xGFPp_FGF1E_LPETGG and 3xGFPp_FGF1E_LPET_MMAE accumulated at the cell surface, as expected (Figure 5C). After 5 min of incubation of cells with 3xGFPp_FGF1E_LPETGG and 3xGFPp_FGF1E_LPET_MMAE at 37 °C, we detected an intracellular GFP signal colocalizing with Rab5a-RFP. The cell surface GFP signal decreased over time, with a concomitant increase in the intracellular signal of GFP until 45 min, when no cell surface staining of 3xGFPp_FGF1E_LPETGG and 3xGFPp_FGF1E_LPET_MMAE was detected (Figure 5C).

We also monitored the kinetics of FGFR1 degradation upon stimulation of U2OS-R1 cells with the trimeric 3xGFPp_FGF1E_LPETGG and its cytotoxic conjugate in the presence of cycloheximide, which blocks the synthesis of new FGFR1 molecules. Changes in FGFR1 levels over time upon stimulation with 3xGFPp_FGF1E_LPETGG and 3xGFPp_FGF1E_LPET_MMAE were analyzed using Western blotting and served as an indicator of lysosomal delivery of the studied molecules. We observed accelerated FGFR1 degradation for both 3xGFPp_FGF1E_LPETGG and 3xGFPp_FGF1E_LPET_MMAE in relation to the wild-type FGF1. Whereas substantial degradation of FGFR1 was detected after 3 h of cell stimulation with the wild-type FGF1, a similar level of FGFR1 degradation was observed already after 1 h of cell treatment with 3xGFPp_FGF1E_LPETGG and 3xGFPp_FGF1E_LPET_MMAE (Figure 5D).

All these data confirm the applicability of GFPp fluorescence for tracking of the trimeric 3xGFPp_FGF1E_LPET_MMAE conjugate. Our data suggest that the cytotoxic conjugate 3xGFPp_FGF1E_LPET_MMAE is highly efficiently and selectively taken up by cells via FGFR1-dependent endocytosis. Furthermore, our data indicate that the multivalency of 3xGFPp_FGF1E_LPET_MMAE may facilitate lysosomal delivery of the conjugate.

### 3.6 High Cytotoxicity of the Trimeric Conjugate

**Figure 6:**
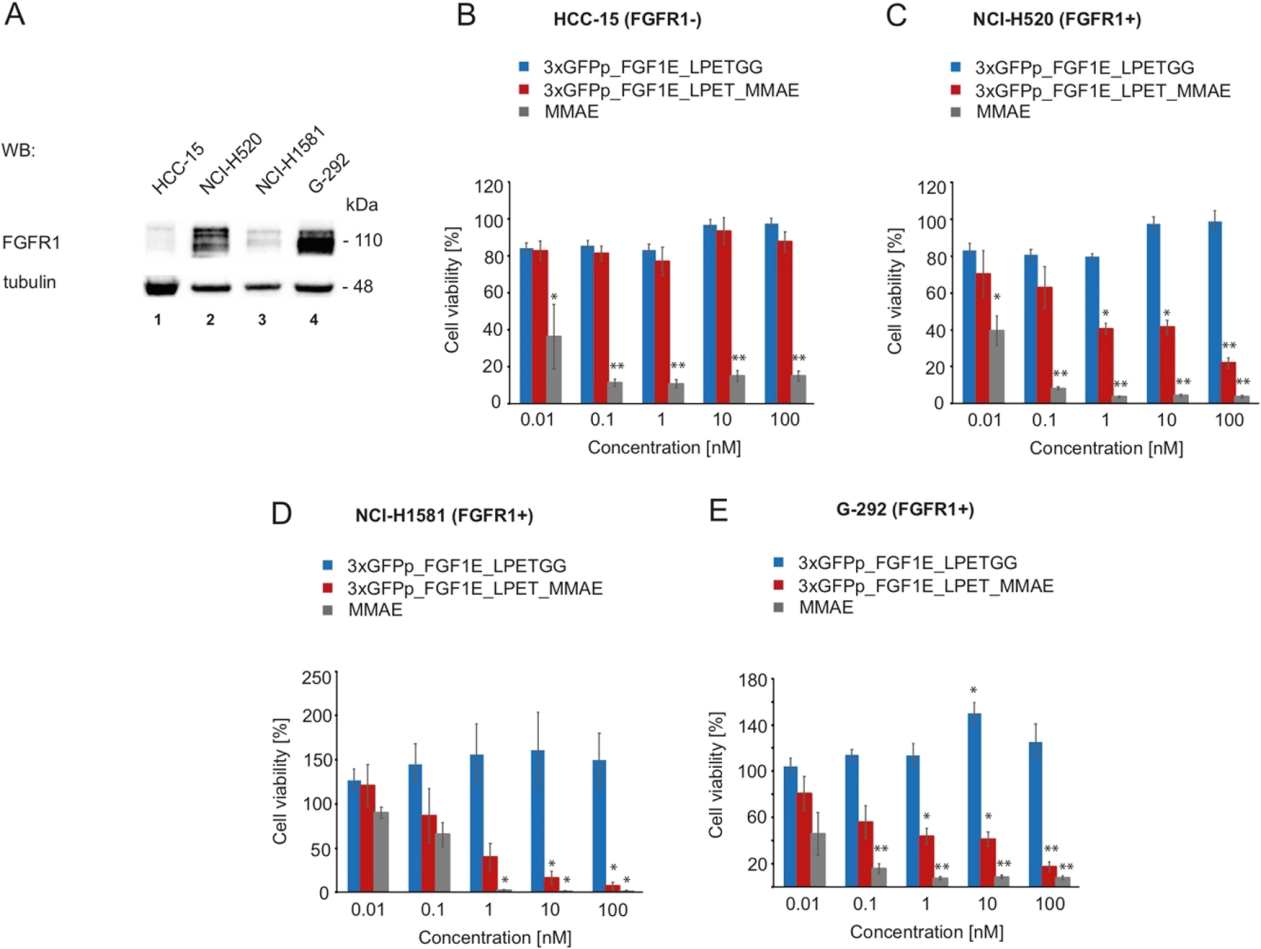
Cytotoxicity of the trimeric GFPp_FGF1E_LPET_MMAE against FGFR1-producing cells. (A) The expression level of FGFR1 in all tested cell lines was analyzed with Western blotting. The level of tubulin served as a loading control. (B–E) Cytotoxicity of the trimeric GFPp_FGF1E_LPET_MMAE. FGFR1-negative cells: HCC-15 (B) and FGFR1-positive cells: NCI-H520 (C), NCI-H1581 (D) and G-292 (E) were treated with increasing concentrations of the conjugate, unconjugated 3xGFPp_FGF1E_LPETGG, or free MMAE for 96 h, and their viability was assessed with the PrestoBlue assay. The data shown are mean values of three independent experiments ± SD. The Student *t*-test was applied for statistical analysis; *p < 0.05 and ^**^p < 0.005.

To determine the cytotoxic potency and FGFR1 selectivity of the 3xGFPp_FGF1E_LPET_MMAE conjugate, we used a panel of cancer cell lines with different levels of FGFR1 expression. As a negative control, we employed the squamous cell lung carcinoma HCC-15 cell line devoid of detectable FGFR1. We used several FGFR1-positive cell lines: lung squamous cell carcinoma NCI-H520, lung large cell carcinoma NCI-H1581, and the osteosarcoma cell line G-292. The expression level of FGFR1 in all tested cell lines was analyzed with Western blotting (Figure 6A).

Each cell line was treated with increasing concentrations of the 3xGFPp_FGF1E_LPET_MMAE conjugate, unconjugated 3xGFPp_FGF1E_LPETGG, and free MMAE as a control. We observed that the unconjugated protein showed no cytotoxicity against all studied cell lines, regardless of the level of FGFR1 expression (Figure 6B–E). The trimeric conjugate 3xGFPp_FGF1E_LPET_MMAE displayed virtually no cytotoxic effect toward the FGFR1-negative HCC-15 cell line (Figure 6B). In contrast, the conjugate exhibited high cytotoxic activity against all FGFR1-positive cell lines tested in a concentration-dependent manner (Figure 6C–E and Table 3). The calculated EC_50_ values of the trimeric conjugate for FGFR1-positive cell lines were in the low nanomolar (for NCI-H520) or even subnanomolar range (for NCI-H1581 and G-292) (Table 3).

**Table 3:** Cytotoxicity of the Conjugate and the Free Drug in Different Cell Lines^a^

EC_50_ [nM]		
cell line	3xGFPp_FGF1E_LPET_MMAE	MMAE
HCC-15	519 ± 124	6 × 10^–3^ ± 3.91 × 10^–4^
NCI-H520	2 ± 5	7.6 × 10^–3^ ± 3 × 10^–4^
NCI-H1581	0.21 ± 0.03	0.15 ± 0.03
G-292	0.61 ± 0.42	8.3 × 10^–3^ ± 6.6 × 10^–4^

a EC_50_ values of GFPp_FGF1E_LPET_MMAE and free MMAE were calculated from Hill’s equation using Origin 7 software (Northampton, MA). Mean values from three experiments ± SD are shown.

These data indicate that 3xGFPp_FGF1E_LPET_MMAE is a trackable, FGFR1-selective, highly potent, and fluorogenic cytotoxic conjugate against FGFR1-overproducing cancer cells.

## 4 Discussion

The development of effective anticancer therapies is still a major challenge in modern medicine. The main difficulty is an effective and precise delivery of cytotoxic drugs into the tumor while avoiding healthy cells and minimizing the side effects of therapy. Currently, one of the most promising strategies in cancer treatment is targeted therapy with cytotoxic conjugates.^3−5^ The aim of this selective therapy is to precisely deliver drugs into the tumor by targeting cancer-specific marker molecules.^1,5,45^ These include cell surface antigens, growth factor receptors, cell adhesion molecules, cytokine receptors, Fas/Fas-ligand molecules, and others.^8,46^

In the targeted therapy approach, monoclonal antibodies, antibody fragments, and receptor ligands, which recognize cancer marker proteins, serve as drug-targeting vehicles.^7,8,47^ Delivery of cytotoxic drugs conjugated to targeting molecules increases the local drug concentration in the tumor vicinity and inside cancer cells, allowing for high selectivity and cytotoxicity at low drug concentrations and minimizing side effects.^45,48^ Such targeting molecules, when properly functionalized, can also serve as molecular probes for tumor imaging. Fluorescent targeting molecules may be helpful in understanding the mechanisms of cellular uptake and action of drugs in the targeted therapy.^45,46^ These conjugates enable real-time monitoring of drug delivery and distribution, as well as therapeutic response, both *in vitro* and *in vivo*. This approach provides direct information on drug accumulation in the tumor and possible undesirable accumulation in healthy tissues.^47^ Importantly, the fluorescence of the conjugate allows for intracellular tracking of the conjugate, so the efficiency and the mechanism of the conjugate internalization into cancer cells and its subsequent intracellular sorting can be monitored.^49^ To date, several studies have confirmed the effectiveness of fluorescent targeting molecules and their conjugates in selective recognition of cancer cells and in monitoring their activity.^50,51^

Since the efficacy of the targeted therapy with cytotoxic conjugates largely depends on the properties of the targeting molecules, our aim was to develop a highly stable, high-affinity, and efficiently internalizing targeting molecule with an intrinsic fluorescence allowing for its tracking. As FGFR1 is overexpressed in various types of cancer, we decided to engineer a targeting molecule specific for this receptor.^13−16^ We took advantage of our recent findings, demonstrating that FGFR1 clustering either with multivalent antibodies or oligomeric ligands enhances the efficiency and alters the mechanisms of FGFR1-mediated endocytosis.^30−32,52^ Additionally, we have shown that oligomerization of FGF1, a natural FGFR1 ligand, constitutes an attractive tool to increase its affinity for the receptor.^30,52^ Inspired by these findings, we have recently demonstrated that the streptavidin-based controlled oligomerization of cytotoxic conjugates targeting FGFR1 and HER2 receptors improves their cellular uptake and cytotoxicity.^52^

Here, we decided to combine these highly desirable features and further functionalize FGFR1-targeting molecules with fluorescence to enable their visualization. We developed a novel, highly efficient system to generate intrinsically fluorescent, self-assembling oligomeric drug carriers targeting FGFR1. We employed FGF1 as an FGFR1-targeting molecule and fluorescent GFPpolygons as a scaffold for controlled oligomerization of the FGFR1 ligand.^33^ Oligomeric GFPp_FGF1 variants display largely enhanced affinity for FGFR1 as compared to the monomeric ligand. We have previously obtained similar results for multivalent antibodies and coiled-coil-triggered FGF1 oligomers.^30,31^ Since the high affinity of the targeting molecules to FGFR1 ensures its precise recognition on the surface of cancer cells, we decided to evaluate the applicability of GFPp_FGF1 oligomers as fluorescent drug carriers in the selective destruction of FGFR1-overproducing cancer cells.

Based on the largely improved binding of trimeric GFPp_FGF1 to FGFR1 and the ease of its isolation, we decided to construct a trimeric cytotoxic conjugate. We employed a highly stable mutant of FGF1, FGF1E, and conjugated it with MMAE via sortase A-mediated ligation in a site-specific manner. The resulting fluorogenic trimeric cytotoxic conjugate 3xGFPp_FGF1E_LPET_MMAE displayed very high stability and high affinity for FGFR1. We made use of the 3xGFPp_FGF1E_LPET_MMAE intrinsic fluorescence, and by using fluorescence microscopy, we have shown that the conjugate is efficiently and selectively internalized into FGFR1-expressing cells. The trimeric 3xGFPp_FGF1E_LPET_MMAE conjugate displays high cytotoxicity against FGFR1-producing cells while remaining neutral toward FGFR1-negative cells. Importantly, its cytotoxicity is one of the highest (EC_50_ in the subnanomolar range) among the conjugates targeting FGFR1 described to date.^25,26,28,53^ For comparison, monomeric conjugates composed of FGF1 and MMAE display much higher EC_50_ values (50–150 nM, depending on the cell line).^25,52^ These data suggest that the oligomerization of the targeting molecules with GFPp scaffolds not only improves the selective delivery of cytotoxic drugs to cancer cells but also allows for monitoring the distribution and intracellular trafficking of the conjugate.

Importantly, our strategy for the development of oligomeric, fluorescent cytotoxic conjugates can be easily adapted to other cancer-specific cell surface molecules. There are numerous cancer markers explored as targets in anticancer therapies, and producing oligomeric, fluorescent conjugates, selective for a particular tumor marker using the approach presented in this study, may increase the effectiveness of the therapy and enable monitoring of conjugate transport.^46,54^ Additionally, our system can be easily modified to allow for the simultaneous attachment of several drugs with a different mode of action. This approach facilitates overcoming the challenges of cancer drug resistance.^55^ The efficacy of the double- or multiwarhead conjugates has been confirmed in several studies.^28,37,56−58^

## 5 Conclusions

Summarizing, our data demonstrate that the controlled oligomerization of FGF1 with GFPp leads to oligomeric FGFR1 ligands with desired valence and enhanced affinity for the receptor. We determined that GFPp_FGF1 oligomers can be used as novel, highly effective, and trackable drug delivery vehicles for the selective treatment of FGFR1-overproducing cancer cells. Importantly, the system presented herein can be easily adapted to develop effective oligomeric conjugates targeting other cancer-specific cell surface marker proteins. We have recently demonstrated that conjugates composed of FGF2 and MMAE efficiently eliminated FGFR1-overproducing tumors in the murine model.^59^ Future work should focus on further modification of GFPp_FGF1 oligomers to eliminate their potential immunogenicity, e.g., by site-specific PEGylation or directed mutagenesis of the GFPp scaffold. Afterward, their applicability for *in vivo* tumor imaging and elimination should be assessed.
